# QCM-D characterization of time-dependence of bacterial adhesion

**DOI:** 10.1016/j.tcsw.2019.100024

**Published:** 2019-04-06

**Authors:** Todd E. Alexander, Lindsay D. Lozeau, Terri A. Camesano

**Affiliations:** Department of Chemical Engineering, Worcester Polytechnic Institute, 100 Institute Road, Worcester, MA 01609, United States

**Keywords:** QCM-D, Biofilm, Bacterial adhesion, Modeling

## Abstract

Quartz crystal microbalance with dissipation monitoring (QCM-D) is becoming an increasingly popular technique that can be employed as part of experimental and modeling investigations of bacterial adhesion. The usefulness of QCM-D derives from this technique’s ability to probe binding and interactions under dynamic conditions, in real time. Bacterial adhesion is an important first step in the formation of biofilms, the control of which is relevant to industries that include shipping, water purification, packaging, and biomedical devices. However, many questions remain unanswered in the bacterial adhesion process, despite extensive research in this area. With QCM-D, multiple variables affecting bacterial adhesion can be studied, including the roles of substrate composition, chemical modification, solution ionic strength, environmental temperature, shear conditions, and time. Recent studies demonstrate the utility of QCM-D in developing new bacterial adhesion models and studying different stages of biofilm formation. We provide a review of how QCM-D has been used to study bacterial adhesion at stages ranging from the first step of bacterial adhesion to mature biofilms, and how QCM-D studies are being used to promote the development of solutions to biofilm formation.

## 1 Introduction

Bacterial adhesion is an important step in biofilm formation, which needs to be further understood in order to develop novel solutions to promote or prevent biofilm formation (Meireles et al., 2015, Tandogan et al., 2017). Undesired biofilm formation causes billions of dollars in economic damage annually in the US and affects a wide variety of sectors, including but not limited to shipping, water treatment (e.g. water supply piping, membranes for water purification, irrigation systems), and healthcare. In the healthcare space alone, infections cause over $45 billion dollars of direct economic damage a year, with a total cost of up to $94 billion (Römling et al., 2014, Scott, 2009). In a 2015 Homeland Security report, the cost of marine biofouling was estimated to be $120 billion dollars annually in the US (McClay et al., 2015). A better understanding of the biofilm formation process is needed to help mitigate its economic effects and provide better outcomes for patients, industrial applications, and homeland security.

Bacterial adhesion to a surface is an extremely complex process that begins with: 1) extracellular and intracellular signaling to either recognize a surface or recruit other bacteria to the surface (Khelissa et al., 2017, Tarnapolsky and Freger, 2018), 2) reversible adhesion to the surface via surface proteins such as fibrils and lipopolysaccharide (LPS) (Goulter et al., 2009, Kostakioti et al., 2013), 3) irreversible adhesion to the surface, 4) Extracellular matrix (ECM) production and growth of the biofilm changes in protein synthesis and morphological changes (Goulter et al., 2009, Khelissa et al., 2017, Kostakioti et al., 2013) and 5) maturation of the biofilm and dispersion (Flemming et al., 2016, Gutman et al., 2013, Kwon et al., 2006, Römling et al., 2014, Tandogan et al., 2017, Tuson and Weibel, 2013, van der Westen et al., 2017).

The initial step of bacterial adhesion is affected by a number of factors, including external factors like surface energy and topography, and strain specific factors, such as fimbriae and other surface proteins (Kostakioti et al., 2013, Römling et al., 2014, Tuson and Weibel, 2013). Not all biofilms are undesired, and in certain applications, such as wastewater treatment and fluidized bed bioreactors, the immobilization of bacteria on surfaces is important for the success of the industrial application (Meireles et al., 2015, Tandogan et al., 2017). Top fed biofilm reactors have been successfully scaled in order to produce acetic acid for example (Qureshi et al., 2005). More studies are needed before we can rationally design surfaces to either prevent or promote bacterial adhesion. These studies often have limitations due to the techniques used to perform them; therefore, new techniques and methods such as the Quartz Crystal Microbalance with dissipation monitoring (QCM-D) can be used to better understand the bacterial adhesion process.

### 1.1 Techniques to measure and characterize bacterial adhesion

Some of the primary techniques that have been used to study bacterial adhesion include atomic force microscopy (AFM), scanning electron microscopy (SEM), flow cells, colony counting, and microcopy (Tandogan et al., 2017). There are many other techniques utilized to study bacterial adhesion, but only a few of the major techniques will be discussed in this review. A more comprehensive list of techniques can be found in Tandogan et al. (2017) and in Meireles et al. (2015).

AFM has been very useful in advancing the study of bacterial adhesion as this technique helps provide direct information about adhesive forces and their strength. (Camesano et al., 2007, Marcotte and Tabrizian, 2008, Strauss et al., 2010, Strauss et al., 2009, Tarnapolsky and Freger, 2018). The AFM tip can be functionalized in order to explore the adhesive forces of different functional groups and coatings. For example, AFM was used to determine the effect of LPS length on bacterial adhesion strength (Strauss et al., 2009). In another study, *S. epidermidis* was coupled directly to the AFM tip, and adhesion forces between the bacteria and self-assembled monolayers (SAMs) was characterized (Liu et al., 2008). This study showed that *S. epidermidis* adhesion was reduced on the SAM surfaces when fibronectin was introduced as a foulant adhesive forces between the bacteria and the fouled SAM surfaces increased. Some limitations of AFM are that the AFM tip needs to be revalidated before each test to ensure there is no contamination, and the technique is low throughput (Liu et al., 2008). However, there are recent improvements in methodology in this area. For example, Formosa-Dague et al. have suggested methods to immobilize an array of living bacteria, which is an improvement over earlier AFM protocols involving bacteria. In addition, these authors discuss how to improve the throughput and statistical relevance of AFM measurements (Formosa-Dague et al., 2015).

Transmission electron microscopy (TEM) and SEM can offer valuable insight into bacterial adhesion processes, especially in combination with other techniques. In most forms of SEM and TEM, which require an ultrahigh vacuum, samples cannot be reused and the cells need to undergo extensive preparation protocols (Meireles et al., 2015). Environmental scanning electron microscopy (ESEM) overcomes some of the challenges of the traditional SEM by allowing for the imaging of wet sample without the need for a conductive coating (Stokes, 2003, Tandogan et al., 2017). The drawback of ESEM is that the resolution is lower than SEM and often the sample still needs to be coated with a conductive material (Stokes, 2003, Tandogan et al., 2017). Light based microscopy techniques are attractive because they are simple, fast and inexpensive (Meireles et al., 2015, Olsson et al., 2009, Olsson et al., 2010). However, their use is limited to transparent surfaces (Meireles et al., 2015).

One reason to look for another methodology to complement the work being done in AFM and other types of microscopy is that time is an important factor in the bacterial adhesion and biofilm formation processes, but this variable can be difficult to incorporate into static microscopy techniques. Biofilm maturation is dynamic and heavily affected by environmental conditions such as shear forces (Chen et al., 2010, Marcotte and Tabrizian, 2008).

QCM-D is able to overcome these shortcomings as it is flow cell technique, which allows for real-time observation of the biofilm formation process (Otto et al., 1999, Tarnapolsky and Freger, 2018). Variables that are difficult to control or change with other techniques, such as flowrate, temperature, ionic strength and nutrient concentration, are easily varied via the QCM-D itself or through the solutions used, offering additional advantages for studying biofilms (Otto et al., 1999, Tarnapolsky and Freger, 2018).

### 1.2 Strategies to prevent bacterial adhesion and biofilm formation

**Fig. 1:**
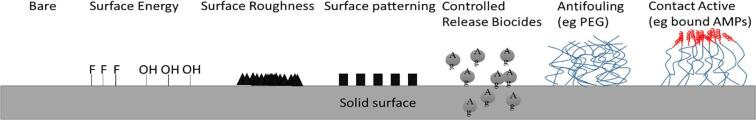
Examples of antibacterial and anti-biofilm surface solutions (Adlhart et al., 2018, Chen et al., 2013, Lozeau et al., 2015). These include chemical or physical modifications to alter surface energy, surface roughness, surface patterning, controlled release of biocides (such as silver), antifouling polymers, and contact-active surfaces (bound antimicrobial peptides (AMPs).

Many strategies (Fig. 1) have been developed to prevent the initial step in biofilm formation, including anti-fouling coatings, contact-active surfaces, increased surface roughness, surface patterning, and biocides (Adlhart et al., 2018, Chen et al., 2013, Lozeau et al., 2015).

The above strategies can be combined for greater and longer term effects, such as the combination of an antifouling polymer with an antimicrobial compound (Adlhart et al., 2018). Types of chemical modifications to a surface include antiadhesive coatings, such as zwitterionic polymers, polyethylene glycol (PEG), hydrogels, and even superhydrophobic coatings, such as slippery liquid infused porous surfaces (SLIPS) (Adlhart et al., 2018, Chen et al., 2013, Lozeau et al., 2015). These strategies work based on changing the surface energy to discourage protein and bacterial adhesion (Banerjee et al., 2011, Damodaran and Murthy, 2016). In the case of PEG-based coatings, it is theorized that there may be a thermodynamic reason for protein and bacterial repellence due to the preference of PEG to complex with water molecules (Banerjee et al., 2011, Damodaran and Murthy, 2016). This strategy can be effective; however, a perfect coverage is needed to prevent fouling and long-term stability, which is a large manufacturing issue (Banerjee et al., 2011, Chung et al., 2007, Graham and Cady, 2014). Contact-active surfaces, which kill bacteria upon interaction, such as quaternary ammonia salts or antimicrobial peptides (AMPs) have shown efficacy (Adlhart et al., 2018, Gao et al., 2012, Glinel et al., 2012, Lozeau et al., 2015). With contact-active surfaces, the challenge of the formation of a conditioning layer by dead bacteria, rendering the surface infective, has not been sufficiently addressed (Adlhart et al., 2018, Gao et al., 2012, Glinel et al., 2012). One strategy to counteract this effect is to combine antifouling polymers with other methods, such as hydrolysis of top layers (Cheng et al., 2008). This can be overcome by coupling with antifouling polymers or via other mechanisms such as hydrolysis of top layers (Cheng et al., 2008). Cheng et al. (2008) used a ‘switchable’ polymer that has a cationic mechanism of action, but then due to hydrolysis of betaine esters between the quaternary amine and the carboxyl, the polymer transitions from a highly cationic charged molecule to a zwitterionic polymer, which causes the release of the dead bacteria (Cheng et al., 2008).

Other chemical-based surface modification strategies involve the release of biocides, such as silver, but this is limited by the reservoir and diffusion profile of the active ingredient (Adlhart et al., 2018, Bonfill et al., 2017, Gao et al., 2012, Pickard et al., 2012, Tiller, 2010). Controlled release has to be used carefully for systems that use traditional antibiotics, as the release itself may promote bacterial resistance (Adlhart et al., 2018, Pickard et al., 2012). In addition, some of the antibacterial compounds in use (such as copper in the shipping industry) have negative environmental impacts (McClay et al., 2015, O’Neill, 2014).

Introduction of surface roughness and patterning can be used to control bacterial adhesion (Perera-Costa et al., 2014, Song et al., 2015a, Vasudevan et al., 2014). This strategy is commonly found in nature and can be applied to various materials, including polymers and metals (Scardino and de Nys, 2011). Feature height and shape have a significant effect on bacterial adhesion; for example, the wing of the clanger cicada has nanoscale pillars (200 nm in height and 60 nm in diameter) that kill bacteria (Perera-Costa et al., 2014, Song et al., 2015a, Vasudevan et al., 2014). Sharklet™ is one example of a patterned surface based on the topography of shark skin to prevent bacterial adhesion, however, long term efficacy and manufacturability are still being optimized (Chung et al., 2007, Graham and Cady, 2014). Due to the diversity of anti-biofouling strategies, there needs to be a way to evaluate how they function in the short- and long-term, as well as to test their application in real-world conditions.

## 2 Factors affecting bacterial adhesion and biofilm maturation evaluated using QCM-D

### 2.1 QCM-D technique and sensitivity

**Fig. 2:**
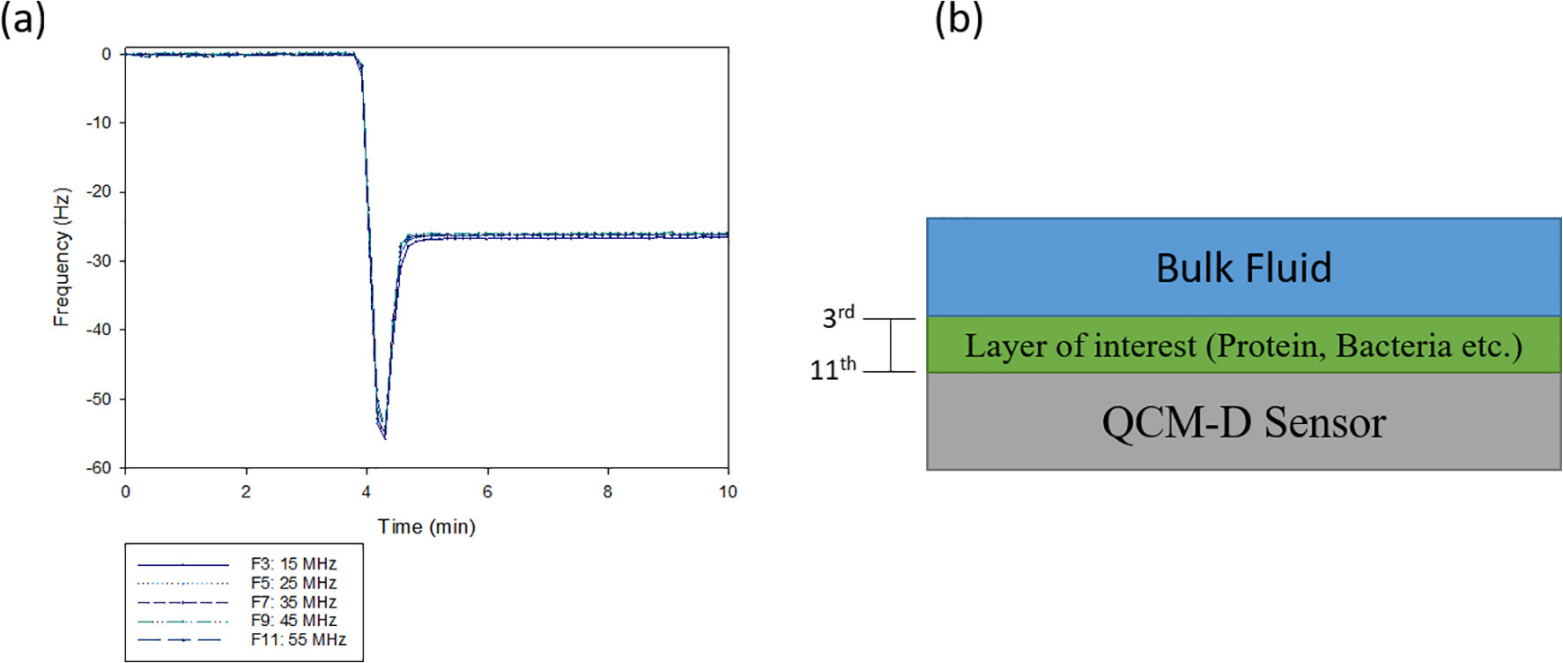
(a) Representative change in frequency for a lipid bilayer adhering to the sensor surface, similar overtone measurement mean similar characteristic structure across the bilayer. The y axis is frequency in hertz and the x axis is time in minutes. Five different overtones are plotted 3rd-11th. (b) Representative image of the penetration depth of various overtones. Higher overtones are more reflective of processes happing near the sensor surface, while lower overtones are representative of processes further from the sensor surface (Mechler et al., 2007).

QCM-D is a non-destructive flow technique that uses an oscillating piezoelectric quartz crystal sensor to measures changes in frequency (Δ*f*) and dissipation (ΔD) in real time (Busscher et al., 2010, Lozeau et al., 2015, Tarnapolsky and Freger, 2018). Recent advances focus on the use of QCM-D to provide more detailed information on bacterial adhesion and interactions with surfaces, under dynamic flow conditions. Variables affecting bacterial adhesion, including the roles of substrate composition, chemical modification, solution ionic strength, environmental temperature, and time can be studied using QCM-D. The QCM-D is a very sensitive technique with the ability to detect mass changes as small as 0.5 ng (Strauss et al., 2009). Additionally, the viscoelasticity of the adhering protein, chemical film or bacteria can be determined due to the ability of the QCM-D to detect and record energetic losses in the form of dissipation. Overtones are frequencies of the QCM-D sensor that are higher than the fundamental frequency, 5 MHz for AT cut silicone dioxide based sensors, that allow for the interpretation of different phenomena at different energies and penetration depths (Goka et al., 2000). Multiple overtones can be measured at once, typically the 3rd through the 11th overtone, with higher overtones measuring processes that are happening closer to the sensor surface (Fig. 2). Changes in frequency (Δf) are related to changes in mass (Δm) and changes in dissipation are related to the rigidity of the film on the surface. This technique allows for real-time analysis, which therefore allows for the study of processes that happen quickly.

Numerous studies demonstrate the power of QCM-D in developing new bacterial adhesion models and studying different stages of biofilm formation. Of particular interest is the ability of the QCM-D to identify time-dependent changes in bacterial attachment and morphological changes (Contreras et al., 2011, Eichler et al., 2011, Feldötö et al., 2008, Gutman et al., 2013, Leino et al., 2011, Marcus et al., 2012, Olsson et al., 2015, Olsson et al., 2011, Otto et al., 1999, Poitras and Tufenkji, 2009, Schofield et al., 2007, Speight and Cooper, 2012, Sweity et al., 2011, Tarnapolsky and Freger, 2018, van der Westen et al., 2017, Zhou et al., 2000). The sensitivity of the QCM-D allows for the detection of changes that happen in seconds or minutes (Olsson et al., 2010), but QCM-D can also be used for very long time scales, even up to days or weeks (Chen et al., 2010, Nivens et al., 1993, Reipa et al., 2006).

### 2.2 Studies of bacterial adhesion factors using QCM-D

QCM-D experiments can be performed under flow or no-flow conditions, and even a combination of the two (Schofield et al., 2007). In addition, due to the non-destructive nature of the QCM-D, experiments can be combined with other techniques, including destructive ones, to gather more information than would not be possible if destructive techniques such as SEM were used alone (Gutman et al., 2013, Leino et al., 2011, Marcus et al., 2012, Olofsson et al., 2005, Olsson et al., 2015, Strauss et al., 2009). Leino et al. (2011) used the QCM-D in combination with both AFM and field emission SEM (FESEM) in order to study the adsorption of *Pseudoxanthomonas taiwanesis* onto cellulose and hemicellulose in the presence of polycations (poly(diallyldimethyl) ammonium chloride (pDADMAC) and polyacrylamide (C-PAM). They found that the pDADMAC adsorbed as a rigid layer and the C-PAM adhered as a thick loose layer; however, the QCM-D signal did not proportionally respond to the amount of bacteria that was adhered. Then, Leino et al. (2011) used the AFM and FESEM to directly image the QCM-D sensor which showed that the bacterial cells clusters in “rafts” with large areas with no bacteria in order to supplement their finding on the QCM-D. Since QCM-D is not destructive, there is possibility for its (Leino et al., 2011) combination with other techniques, such as fluorescent microscopy, AFM and SEM (Lozeau et al., 2015, Strauss et al., 2009).

**Fig. 3:**
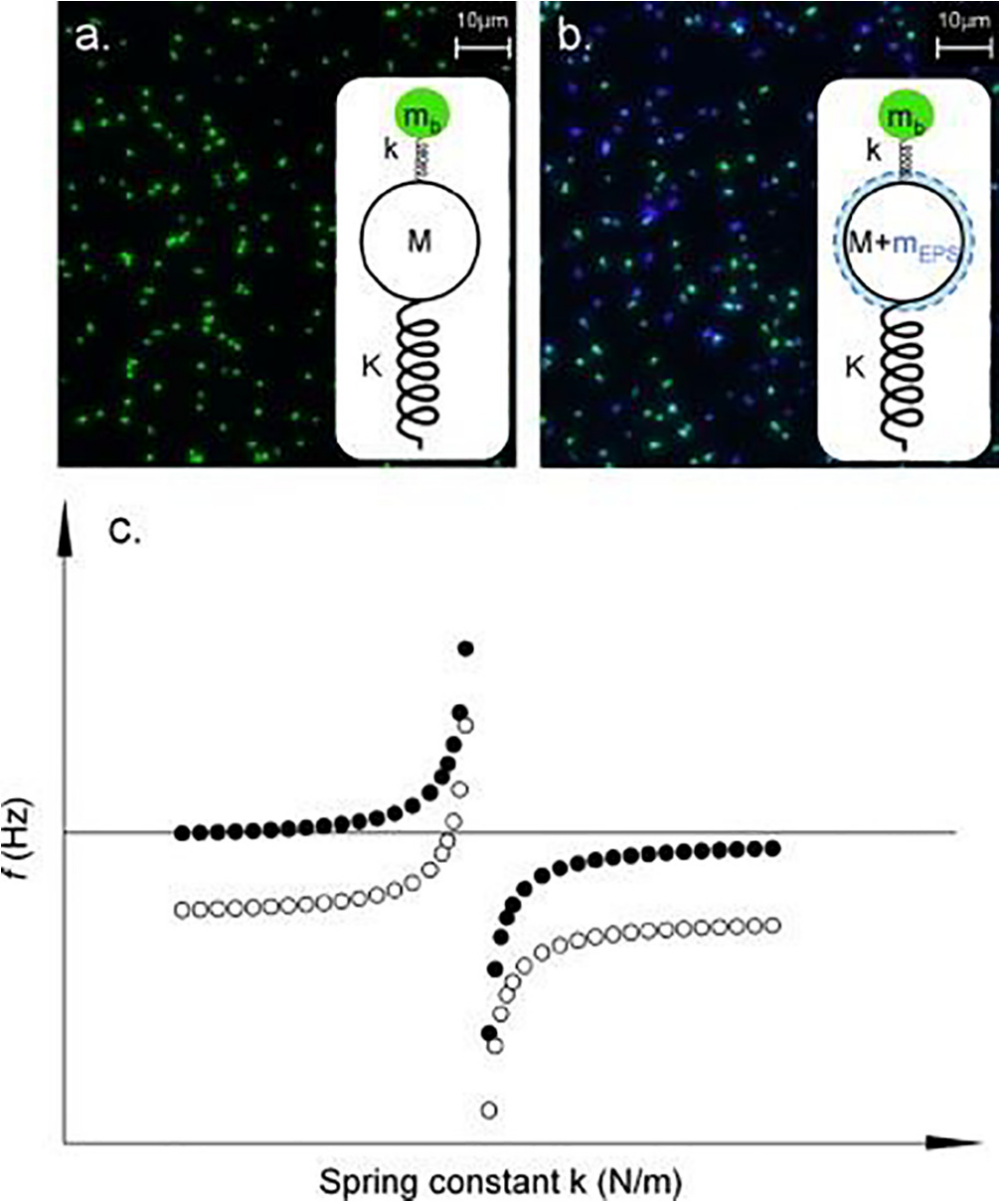
*Reprinted from* Journal of Colloid and Interface Science, 357, Olsson, A. L. J., van der Mei, H. C., Busscher, H. J., Sharma, P. K., Acoustic sensing of the bacterium–substratum interface using QCM-D and the influence of extracellular polymeric substances., 135–138., 2011, with permission from Elsevier. (a) Non-EPS producing strain, (b) EPS producing strain, (c) Maxwell and Voigt-Kelvin Modeling.

An example of a strong combination is to combine QCM-D with fluorescence microscopy. In this case, it is possible to calculate the individual contribution that a cell has on the surface, frequency and dissipation shifts, and thus the strength of adhesion for a single cell (Fig. 3) (Marcus et al., 2012, Olsson et al., 2011). Marcus et al. (2012) used the window module of the QCM-D, combined with fluorescence microscopy, to study the effect of growth phase on the adhesion of *P. aeruginosa* onto hydrophobic and hydrophilic surfaces at the individual cell level. They found that hydrophobic bacteria formed strong elastic bonds with a hydrophobic surface, and that highest dissipation per cell was found with hydrophilic cells on a hydrophilic surface due to a thin liquid gap between the cell and the surface, thus demonstrating the importance of growth phase on adhesion (Marcus et al., 2012)

Understanding the fundamental process by which the bacterium goes from being reversibly attached to irreversibly attached is important for the design of new strategies to prevent bacterial adhesion (Busscher et al., 2010, Contreras et al., 2011, Khelissa et al., 2017, Kostakioti et al., 2013, Leino et al., 2011, Römling et al., 2014, Tandogan et al., 2017). QCM-D is useful for studying the bacterial surface proteins that play a role in the transition from a reversible to irreversible adhesion process (Olsson et al., 2009, Olsson et al., 2010, Otto et al., 1999, Strauss et al., 2009). For example, Olsson et al. (2009) used *Streptococcus salivarius* mutants that had surface appendages of know length in order to study the effect that fimbriae length had on adhesion over time. “Bald” bacteria demonstrated a frequency decrease, however, more fibrillation led to the adherence of more bacteria which is in agreement with other studies (Olofsson et al., 2005, Olsson et al., 2009, Strauss et al., 2010, van der Westen et al., 2017). It was also found that dissipation was linear when the number of cells was normalized but higher for those with longer fibrils. In a 2010 study, Olsson et al. (2010), using the same strain of bacteria, demonstrated that the adhesion went from reversible to irreversible in 55 s.

The QCM-D can also be used for the study of longer term processes in biofilm formation, including the stage of EPS deposition. For example, Olsson et al. (2011) measured the EPS secretion of *S. epidermidis* over time, in the absence of growth media. EPS is a major component of biofilms that helps protect the bacteria from antibiotics and other environmental hazards. This study was one of the first to point out how the QCM-D signal is affected by EPS, and this work was built upon with further models to take into account the role of bacterial surface molecules (Kwon et al., 2006, Olsson et al., 2015, Olsson et al., 2009, Olsson et al., 2011, Sweity et al., 2011). In addition, the ability to probe viscoelastic properties provides more information on biofilm formation. Reipa et al. studied the long term growth of *P. aeruginosa* in tap water and found that even though thickness of the biofilms did not change, their viscoelastic properties changed as the biofilms matured, becoming less dense and more viscoelastic (Reipa et al., 2006). They also found that when nutrients were reduced, the biofilms became more rigid (Reipa et al., 2006). Another study used QCM-D to examine how DNAse 1 and EPS-degrading enzymes break up early stage and mature biofilms (Kostakioti et al., 2013). Combining the different types of information that are provided by QCM-D helps in the development of strategies for biofilm removal, which are known to depend on the stage of biofilm formation (Chen et al., 2013, Gall et al., 2013, Kostakioti et al., 2013, McClay et al., 2015, Simoes et al., 2010).

Molino et al. (2006) used the QCM-D to study the interaction of secreted mucilage of two marine diatoms in order to study the first steps in marine biofouling. Using *Craspedostauros australis* (a weak biofouling diatom) and *Amphora coffeaeformis* (a strong and common biofouling diatom), the authors measured the time-dependent deposition of EPS for both species. By calculating the ratio of the change in frequency to the change in dissipation (Δf/ΔD) for each diatom, they obtained reproducible signatures for each species that were related to their biofouling properties, and used this to suggest antifouling strategies (Molino et al., 2006).

Another interesting application of QCM-D was its use to help determine signaling pathways during bacterial adhesion (Otto and Silhavy, 2002). Using both modified and unmodified *E. coli* strains, Otto and Silhavy (2002) demonstrated the importance of the Cpx-signaling pathway on adhesion. They determined that NIpE, an outer membrane lipoprotein, plays a major role in bacterial adhesion since when it is absent the Cpx-signaling pathway was not triggered. Both the NIpE protein and the Cpx-pathway are needed for bacterial adhesion and when either were compromised bacteria did not adhere well to any of the surfaces tested (Otto and Hermansson, 2004). This was apparent in the frequency and dissipation measurements and in the slope of ΔD/Δf, where the viscoelastic properties of the wild type cell were significantly different than the mutants (Otto and Silhavy, 2002). This can lead to the development of solutions to prevent bacterial adhesion by targeting either the protein of the Cpx-pathway. These studies demonstrate the power of the QCM-D to systematically help determine the signaling pathways via bacterial mutants and the effect of the outside environment, such as hydrophobicity, on bacterial adhesion (Gutman et al., 2013, Olsson et al., 2009, Otto and Hermansson, 2004). This could lead to novel design of anti-biofilm molecules that could be developed in a traditional pharmaceutical setting.

## 3 QCM-D evaluation of modified surfaces that affect bacterial adhesion

With a better understanding of how bacteria adhere to surfaces, there is great interest in using QCM-D, among other techniques, to study and design surfaces that prevent adhesion (Chen et al., 2013, Flemming et al., 2016, Glinel et al., 2012, Khelissa et al., 2017, Kostakioti et al., 2013, Tandogan et al., 2017). Some strategies are based on physical changes to the structure of the surface, for example, patterning (Sharklet™), roughness, and mechanical deformation (Chung et al., 2007, Graham and Cady, 2014). Other methods involve changing the surface energy via modification of the surface through plasma etching or chemically binding polymers such as polyethylene glycol or flouropolymers (Adlhart et al., 2018, Chen et al., 2013, Lozeau et al., 2015). Surfaces can also be modified to allow for direct killing of bacteria through the release of a biocide or a coating of a biocide such as antimicrobial peptides or quaternary ammonia salts (Adlhart et al., 2018, Gao et al., 2012, Glinel et al., 2012, Tiller, 2010). Contreras et al. (2011) used the QCM-D to study the ability of sodium dodecyl sulfate (SDS) to clean various functionalized surfaces and found that there was mixed success in its use at removing conditioning proteins and polysaccharides, BSA and alginate, from the surfaces tested. The ease at which surfaces can be cleaned is also a factor in the design of anti-fouling surfaces.

### 3.1 Passive strategies to prevent bacterial adhesion

Surface patterning is one strategy to prevent or promote bacterial adhesion, and a number of QCM-D studies have examined the effect of patterning on factors that affect bacterial adhesion (Cerf et al., 2009, Qureshi et al., 2005, Welle et al., 2005). For example, Welle et al. (2005) used the QCM-D to study protein adhesion onto patterned polystyrene surfaces. By application of the Voigt-Kelvin viscoelastic model, they determined the amount of protein adsorption and the viscoelastic properties of the film. Cerf et al. (2009) examined the nano-mechanical properties of live and dead bacteria on nano-patterned surfaces using QCM-D and AFM. QCM-D was useful for measuring the electrostatic binding forces and determining which surfaces the bacteria adhered best to. In another study, Thickett et al. (2012) examined the effect of collagen adsorption onto micro-patterned surfaces of two different polymers, polystyrene and poly(*N*-vinylpyrrolidone). From the frequency shift, it was determined that collagen adsorption was much higher on the polystyrene surface, adsorbed viscoelastically (increase in dissipation) on both surfaces, and could be removed via rinsing, as observed by a return in frequency close to the baseline value (Thickett et al., 2012).

For antifouling surfaces such as PEG, zwitterionic polymers, and SAMs, the QCM-D can be useful for characterization deposition and hydration of the film itself, in addition to studying how bacteria interact with the surface. The surface characterizations can be used to directly incorporate variables, including polymer density, hydration, and thickness, to models of bacterial adhesion. For example, Yandi et al. (2014) showed that they could use the QCM-D to study film thickness and hydration level and couple that directly with the effect on bacterial adhesion. The thickness of random poly(HEMA-co-PEG10MA) copolymer brushes affected antifouling behavior directly, and film hydration was a critical component. Reduced antifouling performance was caused by a lower hydration capacity of thin films, and that entanglement and crowding of thicker films reduced hydration capacity (Yandi et al., 2014). Many QCM-D based studies of antifouling substrates focus on protein adhesion as that is often considered to be a primary step before bacterial adhesion (Boulmedais et al., 2004, Contreras et al., 2011, Guo et al., 2018, Marcotte and Tabrizian, 2008, Song et al., 2015b, Welle et al., 2005). Eichler et al. (2011) studied the effectiveness of a dendrimer-based coating to repel bacteria after being conditioned with salivary proteins. They were able to determine the mass of protein that adhered to each film using an extended Voigt model. When the mass of the water adsorption was taken into account, the dendrimer polymers outperformed the other coatings and reduced bacterial adhesion. QCM-D was essential to uncovering this result because this technique allowed the authors to measure both the mass of adsorbed protein and the mass of the adsorbed water layer. As another example, Muszanska et al. (2011) studied a family of triblock copolymers, consisting of a hydrophobic core and hydrophilic end groups. They determined the hydrated thickness and viscoelastic properties of the polymers and demonstrated how the polymers were conformationally adhered to the surface, which each play a role in bacterial adhesion.

### 3.2 Active strategies to prevent bacterial adhesion

Contact active surfaces are an attractive method to prevent biofilm formation, often these strategies employ compounds that are active in solution which make studying their bound properties and mechanisms of action important (Atefyekta et al., 2018, Lozeau et al., 2015). A growing area of research is contact active surfaces based on antimicrobial peptides (AMPs), due to their broad spectrum activity and low likelihood of bacterial resistance (Lozeau et al., 2015). Lozeau et al. (2015) demonstrated the ability of the QCM-D to determine the mechanism of action of a covalently bound antimicrobial peptide chrysophsin-1, and showed how peptide activity was related to the length of the tether molecule. By combining QCM-D with fluorescence microscopy, the authors showed that for the short tether, the key variable affecting activity was the cationic charge of the antimicrobial peptide, making it interact with the membrane. However, for a sufficiently long tether, a pore forming mechanism controlled the interaction between the antimicrobial peptide and the membrane, and this was similar to the behavior of C-CHY1 in solution (Lozeau et al., 2015). Atefyekta et al. (2018) used QCM-D to quantify the mass of tethered peptide on a surface, as well as to study the stability of the bond. Similarly, Yoshinari et al. (2006) used QCM-D with a window module in order to quantify AMP adsorption on PMMA and directly couple that to antimicrobial activity. Etienne et al. (2004) used the QCM-D to study the effect the number of AMP-impregnated polyelectrolyte layers had on the killing of *Micrococcus luteus* and *Escherichia coli,* and determined that 10 layers was optimal compared to 5 or fewer layers.

## 4 Methods and modelling of QCM-D systems: Current status and future directions

The QCM-D is a non-destructive flow technique that uses an oscillating piezoelectric quartz crystal sensor to measures changes in frequency (Δf) and dissipation (ΔD) in real time (Johannsmann, 2008, Sauerbrey, 1959, Steinem and Janshoff, 2007). The changes in frequencies can be related directly to changes in mass through the Sauerbrey equation for rigid films and adhesion. However, these models often fall short when trying to directly measure bacterial adhesion due to the complex processes that are involved in bacterial adhesion, as well as coupled resonance (Tarnapolsky and Freger, 2018, van der Westen et al., 2017). Coupled resonance is most commonly observed when there is a positive shift in frequency is observed unexpectedly when mass is in fact being added to the system. Fimbriae and proteins on the bacterial surface can cause this phenomenon (Tarnapolsky and Freger, 2018). Further, if the inertial and spring elastic force of a bacteria on the surface is perfectly balanced, then Δ*f* will be zero (Tarnapolsky and Freger, 2018). The Voigt-Kelvin extended viscoelastic model corrects the Sauerbrey estimations for higher energy dissipation by adding terms to the Δ*f* relation to mass and ΔD relation to film rigidity (Tarnapolsky and Freger, 2018, van der Westen et al., 2017). Analysis of frequency and dissipation changes at different overtones, corresponding to differing penetration depths (250 nm maximum), allows for the study of the effect of fimbriae length or other bacterial surface molecules on adhesion (Olsson et al., 2009, Olsson et al., 2010, Olsson et al., 2011), which provides for real-time mechanistic study of the bacterial adhesion process (Tarnapolsky and Freger, 2018).

### 4.1 Models based on change in frequency

Many of the models that improve on the Sauerbrey relationships make use of data from multiple overtones, which makes the model output more accurate and informative (Feldötö et al., 2008, Lozeau et al., 2015, Olofsson et al., 2005, Tarnapolsky and Freger, 2018, Yandi et al., 2014). For example, the Johannsmann model improves on the Sauerbrey model for biopolymers, by incorporating multiple overtones, and uses a statistical regression line of each overtone related to mass change in order to determine the overall changes mass (Feldötö et al., 2008, Johannsmann, 2008). New models should include the ability to incorporate different overtones in order to fully use all the information that the QCM-D acquires, temperature, frequency and dissipation separation at different overtones (Feldötö et al., 2008, Tarnapolsky and Freger, 2018). Continued model development is needed to better interpret complex systems studied using the QCM-D (Tarnapolsky and Freger, 2018).

Fundamental changes in frequencies can be related directly to changes in mass through the Sauerbrey equation (1) for rigid films and adhesion (Camesano et al., 2007, Lozeau et al., 2015, Sauerbrey, 1959, Strauss et al., 2009). Where $f_{o}$is the resonant frequency of the sensor, A is the piezoelectrically-active area of the crystal, $\rho_{q}$ is the density of quartz and $\mu_{q}$ shear modulus of quarts for AT-cut crystals.

(1) $$\Delta f=-\frac{2f_{o}^{2}}{A\sqrt{\rho_{q}\mu_{q}}}\Delta m$$

This can be simplified, where C is a constant of 17.8 ng/cm^2^/Hz, where the resonant frequency is 5 MHz, and n is the corresponding overtone number.

(2) $$\Delta m=-\frac{C\cdot \Delta f}{n}$$

The QCM-D also measures the energetic loss, or energy dissipation of the crystal, which is directly related to the viscoelasticity of deposited films. Dissipation (D) can be described by the following equation.

(3) $$D=\frac{G^{\prime \prime }}{2\pi G^{\prime }}$$

Where *G*′ and G″ represent the loss and storage modulus respectively. The Sauerbrey relation is only valid for rigid films where the dissipation is close to zero. For non-rigid films the Sauerbrey relation will often underestimate adhered mass and cannot be used for films with significant dissipation. Bacterial cells are covered with various proteins, sugars and other functional groups which can allow for non-rigid contact/ adhesion with the QCM-D sensor surface. This also causes coupled resonance which can present a positive frequency shift even though there is an addition of mass to the surface.

The Johnnsmann model differs from the Sauerbrey model in that it accounts for viscoelasticity of the adsorbed layer (Feldötö et al., 2008, Johannsmann, 1999). The model can be mathematically represented as:

(4) $$\delta \hat{f}\approx -f_{o}*\frac{1}{\pi \sqrt{\rho_{q}u_{q}}}\left(2\pi f\rho d+\hat{J}\left(f\right)*\frac{{\left(2\pi f\right)}^{3}\rho_{f}^{2}d^{3}}{3}\right)$$

Where $\delta \hat{f}$ is the shift in complex frequency, d is the thickness of the film, f is the is the resonance frequency of the crystal and $\hat{J}\left(f\right)$ is the complex shear compliance. The equivalent mass can be calculated using Eq. (5).

(5) $${\hat{m}}^{*}=m^{o}+\hat{J}\left(f\right)\frac{\rho_{q}{\left(2\pi f\right)}^{2}d^{2}}{3}$$

Where ${\hat{m}}^{*}$ is the equivalent mass and $m^{o}$ is the true sensed mass.

### 4.2 Models based on change in frequency and energetic losses (dissipation)

**Fig. 4:**
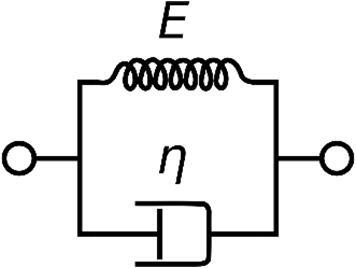
A Voigt-Kelvin material can be represented by a purely elastic spring connected in parallel with a purely vicious dampener. By Pekaje at English Wikipedia (Transferred from en.wikipedia to Commons.) [Public domain], via Wikimedia Commons.

The Voigt-Kelvin extended viscoelastic model corrects the Sauerbrey estimations for higher energy dissipation by adding terms to the Δf relation to mass and ΔD relation to film rigidity(Lozeau et al., 2015, van der Westen et al., 2017). The Voigt-Kelvin-Model cam be represented as a purely elastic spring and a purely viscous dampener in parallel as seen in Fig. 4 and represented mathematically below in Eq. (6).

(6) $$\Delta f+\frac{i\Delta Df_{o}}{2}=\frac{f_{F}m_{p}}{\pi Z_{q}}\cdot N_{p}\left[\frac{\omega_{s}^{3}\left(\omega_{p}^{2}\right)-\left(\gamma^{2}\right)-\omega_{s}\omega_{p}^{4}}{\left(\omega_{s}^{2}\right)-{\left(\omega_{p}^{2}\right)}^{2}+\omega_{s}^{2}\omega_{\gamma }^{2}}\right)+i\left(\frac{\omega_{s}^{4}\gamma }{\left(\omega_{s}^{2}\right)-{\left(\omega_{p}^{2}\right)}^{2}+\omega_{s}^{2}\omega_{\gamma }^{2}}\right]$$

Where $f_{o}$ is the resonant frequency of the sensor, $f_{F}$ is the fundamental frequency of the crystal, $m_{p}$ is the inertial mass of the particle or bacteria, $\omega_{p}$ is the resonance angular frequency for the particle or bacteria, $\omega_{s}$ is the sensor angular frequency, $Z_{q}$ is the acoustic impedance of an AT-cut quartz crystal and $N_{p}$ is the number of adhering particles or bacteria. Where $\gamma =\frac{\xi }{m_{p}}$ and ξ is the drag coefficient.

**Fig. 5:**
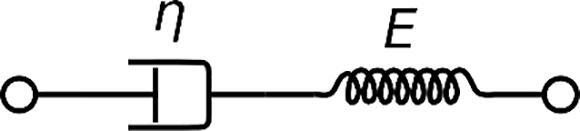
Maxwell representative model. A Maxwell material can be represented by a purely elastic spring connected in series with a purely vicious dampener. By Pekaje at English Wikipedia (Transferred from en.wikipedia to Commons.) [Public domain], via Wikimedia Commons.

Similarly the Maxwell model can be used as an improvement to the Sauerbrey model when dissipation is high (van der Westen et al., 2017). The Maxwell model can be represented as a purely elastic spring and a purely viscous dampener in series as seen in Fig. 5 and represented mathematically below in equation (7) (van der Westen et al., 2017).

(7) $$\Delta f+\frac{i\Delta Df_{o}}{2}=\frac{f_{F}N_{p}}{\pi Z_{q}}\cdot \left[i\omega_{s}m_{p}\left(\frac{1}{1-\frac{\omega_{s}^{2}}{\omega_{p}^{2}}+\frac{i\omega }{\gamma }}\right]\right)$$

It is not clear if the Voigt-Kelvin or Maxwell models are better for studying bacteria with the QCM-D (van der Westen et al., 2017). van der Westen et al. (2017) found that including polydispersity in the models had no effect for “bald” *S. salivarius* HBC12. For the *S. salivarius* HB7 the Maxwell equation led to a mass four times than what was calculated with the Voigt model. While these models can be useful and the output parameters of frequency and dissipation can be used to study bacterial adhesion and biofilm maturation as seen in the studies that have been present. It is clear that better models are needed in order to fully harness the power of the QCM-D. Several groups have attempted to do so (Olsson et al., 2012, Song et al., 2014).

### 4.3 Models based on ΔD/Δf

Many studies are able to use t*h*e ratio of ΔD/Δf in order to gain insight into the bacterial adhesion and biofilm maturation process (Fredriksson et al., 1998, Otto et al., 1999, Sweity et al., 2011, Tarnapolsky and Freger, 2018, Zhou et al., 2000). ΔD/Δf can be used to represent the viscoelastic properties of the deposited bacteria or biofilm (Fredriksson et al., 1998, Otto et al., 1999, Sweity et al., 2011, Tarnapolsky and Freger, 2018, Zhou et al., 2000). For example, Tarnapolsky and Freger (2018) used ΔD/Δf to determine the structure of the biofilm as well as use the ΔD/Δf profile to determine two different biofilm formation regimes. By combining this analysis with other QCM-D outputs this ratio can be used to detect very sensitive processes including conformational changes, for antifouling surfaces, biofilm removal strategies, and restructuring of the bacterial cells or biofilm itself (Feldötö et al., 2008, Kwon et al., 2006, Lozeau et al., 2015, Muszanska et al., 2011, Tarnapolsky and Freger, 2018, Yandi et al., 2014).

However, these models often fall short when trying to directly measure bacterial adhesion due to the complex processes that are involved in bacterial adhesion as well as coupled resonance (Tarnapolsky and Freger, 2018, van der Westen et al., 2017). Due to the various fimbriae and proteins on the bacterial surface coupled resonance may cause a positive shift in frequency which is not what would be expected due to an addition of mass on the sensor surface. This is the major challenge when modeling bacteria with the QCM-D; however, valuable information about adhesion strength and progression, based on the dissipation parameter, can be gleaned and has allowed for new model development.

### 4.4 *Recent models:* Dissipative elastic contributions and frequency with overtone dependence

**Fig. 6:**
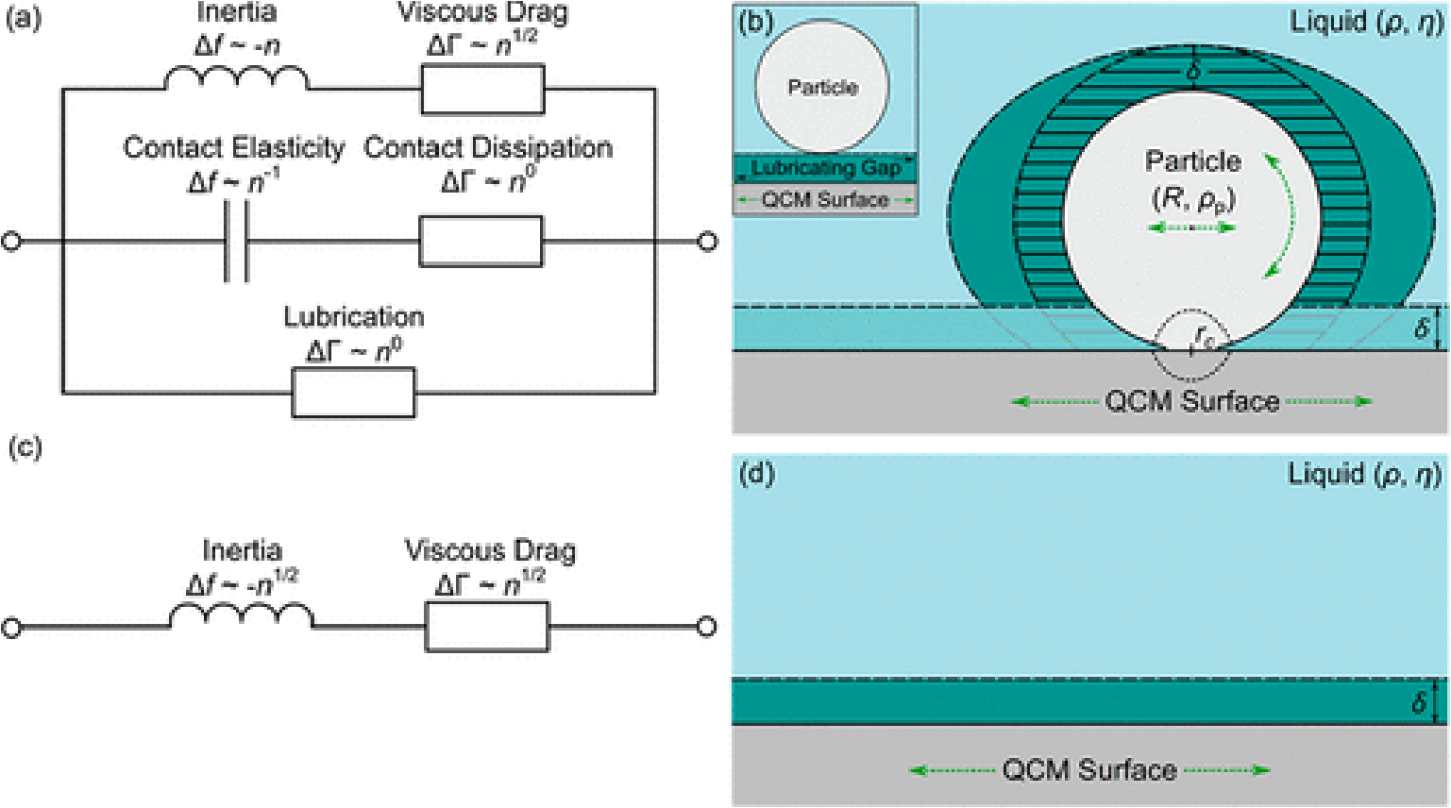
“Reprinted (adapted) with permission from Tarnapolsky and Freger (2018). Modeling QCM-D Response to Deposition and Attachment of Microparticles and Living Cells. Analytical Chemistry. Copyright 2018 American Chemical Society.” The equivalent electrical circuit for the model developed by Tarnapolsky and Freger with (a) and (b) representing a particle or bacteria ahdearing to a crystal and (c) and (d) repsresnting a bare crystal.

Tarnapolsky and Freger (2018) sought out to develop a model for QCM-D by describing the interaction of a freely oscillating sphere in an unbounded fluid. They then tested and validated their model by first using abiotic spheres under various surface chemistry’s and ionic strengths then validated is by using *P. fluorescens.* A graphical representation of the model can be seen in Fig. 6.

Mathematically their model is as follows:

(8) $$Z_{L}^{*}=\frac{\sigma }{\dot{u}}$$

Where $Z_{L}^{*}$is the complex load impedance when a particle contacts the QCM-D sensor surface and σ is the tangential stress and $\dot{u}$ is the tangential velocity at the sensor–solution interface (Tarnapolsky and Freger, 2018). Frequency of a given overtone is related to $Z_{L}^{*}$ by the following equation:

(9) $$\Delta f_{n}=-\frac{f_{F}}{\pi Z_{q}}Im\left(\Delta Z_{L}^{*}\right),\Delta \Gamma_{n}=\frac{f_{F}\eta }{2}\Delta D_{n}=\frac{f_{F}}{\pi Z_{q}}Re\left(\Delta Z_{L}^{*}\right)$$

Where ${\Delta f}_{n}$ is the frequency, ${\Delta \Gamma }_{n}$ is the bandwidth and ${\Delta D}_{n}$ is the dissipation shift shifts for overtone *n*, and *f*_F_ = 5 MHz is the fundamental frequency, and *Z*_q_ = 8.8 × 10^6^ kg m^−2^ s^−1^ is the acoustic impedance of the AT-cut quartz sensor (Tarnapolsky and Freger, 2018). Further derivation and simplification can be found in Tarnapolsky et al. (Tarnapolsky and Freger).

Tarnapolsky and Freger (2018) were able to successfully develop a model that incorporates both dissipative and elastic contributions and frequency with overtone dependence. More importantly this allow for the direct study of dissipation due to contact of the cell with the surface, eliminating dissipation due to bulk bacterial motion under flow. This allows for better analysis of both bacterial adhesion and biofilm analysis in future studies. While this new model is promising further validation under more conditions, different surfaces, temperature and bacterial strains must be tested. Additional models will likely be necessary for conditions under different use techniques such as those for testing antifouling and antimicrobial surfaces.

## 5 Conclusions/outlooks

The QCM-D is a powerful technique that allows for the study of bacterial interactions with surfaces under many types of conditions. Given the cost of unwanted biofilms in various industries and rising antibiotic resistance, it is clear that there is a need to better understand the biofilm formation process in order to develop strategies for their control. A number of variables that include temperature, pH, ionic strength, and surface chemistry are easily changed and manipulated using the QCM-D, either through experimental design or via pre and post treatments of the sensor surface. Additionally, since this technique is nondestructive, it can be combined readily with other techniques for more powerful and in depth analysis. QCM-D can give information at scales that range from the single cell level to a biofilm level. The most important advantages are the range of properties that can be measured (viscoelasticity, conformational changes), and the real-time analysis with a resolution of less than 1 s.

Data analysis is critical to interpretation of the phenomena happening on the surface of the sensor of the QCM-D, thus it is important to use the proper model for each experimental situation. The Sauerbrey equation can be used for studies that examine the adhesion of various conditioning proteins onto rigid surfaces where the proteins of interest do not adsorb significant amount of water (are not viscoelastic). Factors such as ionic strength, pH, temperature and hydrophobicity can be studied. The Voigt-Kelvin viscoelastic and Maxwell models allow for the study of more viscoelastic and complex systems and are therefore, appropriate to use when studying antimicrobial and anti-adhesive surfaces. Studies of well-defined bacteria, such as those with known fibril lengths, can also be studied using the Voigt-Kelvin viscoelastic and Maxwell models, with Maxwell modelling more appropriate for more fluid-like viscoelastic materials (Voinova et al., 1999). Additionally, Δf/ΔD and ΔD/Δfcombined with careful overtone analysis can yield deep insights the properties of bacterial adhesion, biofilm maturity, distance from the surface and even signature profiles (Fredriksson et al., 1998, Sweity et al., 2011, Tarnapolsky and Freger, 2018). The QCM-D alone can yield powerful insights but it can be used in combination with other technique due to its non-destructive nature to yield even more power results. New models such as the one developed by Tarnapolsky and Freger (2018) allow for more powerful insight and can allow for more standalone QCM-D studies however it still needs more validation. In the meantime other model should continue to be developed and ideally a general model that is sufficiently complete but simple can be developed and adopted. With further model development the QCM-D has the ability to be a major and important tool in the development of the next generation of industrial, medical and biomaterials.
